# Opto-Epigenetic Regulation of Histone Arginine Asymmetric Dimethylation via Type I Protein Arginine Methyltransferase Inhibition

**DOI:** 10.1021/acs.jmedchem.4c02199

**Published:** 2025-02-17

**Authors:** Shuting Xu, Kaiqi Long, Tianyi Wang, Yangyang Zhu, Yunjiao Zhang, Weiping Wang

**Affiliations:** † State Key Laboratory of Pharmaceutical Biotechnology, The University of Hong Kong, Hong Kong 999077, China; ‡ Department of Pharmacology and Pharmacy, Li Ka Shing Faculty of Medicine, The University of Hong Kong, Hong Kong 999077, China; § Laboratory of Molecular Engineering and Nanomedicine, Dr. Li Dak-Sum Research Centre, The University of Hong Kong, Hong Kong 999077, China; ∥ The Second Affiliated Hospital, School of Medicine, South China University of Technology, Guangzhou 510006, P. R. China; ⊥ School of Biomedical Sciences and Engineering, National Engineering Research Center for Tissue Restoration and Reconstruction and Key Laboratory of Biomedical Engineering of Guangdong Province, South China University of Technology, Guangzhou 510006, P. R. China

## Abstract

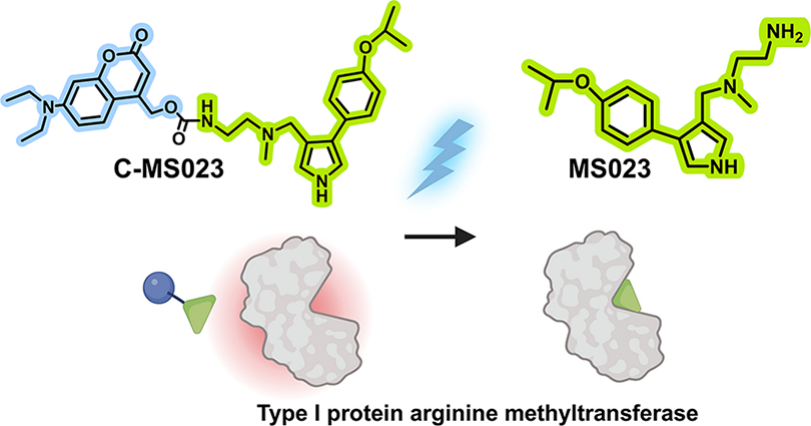

Histone arginine asymmetric dimethylation, which is mainly catalyzed by type I protein arginine methyltransferases (PRMTs), is involved in broad biological and pathological processes. Recently, several type I PRMT inhibitors, such as MS023, have been developed to reverse the histone arginine dimethylation status in tumor cells, but extensive inhibition of type I PRMTs may cause side effects in normal tissues. Herein, we designed a photoactivatable MS023 prodrug (C-MS023) to achieve spatiotemporal inhibition of histone arginine asymmetric dimethylation. In vitro studies showed that C-MS023 exhibited reduced potency in inhibiting type I PRMTs. Importantly, visible light irradiation at 420 nm could trigger the photolysis of the prodrug, thereby liberating MS023 for effective downregulation of histone arginine asymmetric dimethylation and DNA replication-related transcriptomic activities. This opto-epigenetic small-molecule prodrug potentially aids in further research into the pathophysiological functions of type I PRMTs and the development of targeted epigenetic therapeutics.

## 1 Introduction

Histone arginine methylation is a fundamental post-translational modification for regulating chromatin status and gene transcription.^1^ Generally, histone asymmetric dimethylation is a marker of transcriptional activation, despite some exceptions.^2,3^ Arginine asymmetric dimethylation is mainly generated by type I protein arginine methyltransferases (PRMTs), including PRMT1–4, PRMT6, and PRMT8.^4^ These enzymes transfer the methyl group from S-adenosyl-l-methionine (SAM) to the guanidino moieties of specific histone arginine residues.^5^ Type I PRMTs participate in broad physiological processes, including chromosome condensation, pluripotency maintenance, and erythropoiesis.^6−8^ Additionally, the upregulation of type I PRMT activities has been found in multiple carcinogenesis mechanisms and is closely correlated with tumor aggressiveness.^9^ Recently, several type I PRMT inhibitors like MS023 have been developed.^4,10−12^ However, the precise regulation of histone arginine asymmetric dimethylation remains an unsolved, yet appealing task for reprogramming malignant tumors without impacting normal physiological activities.

**Figure 1:**
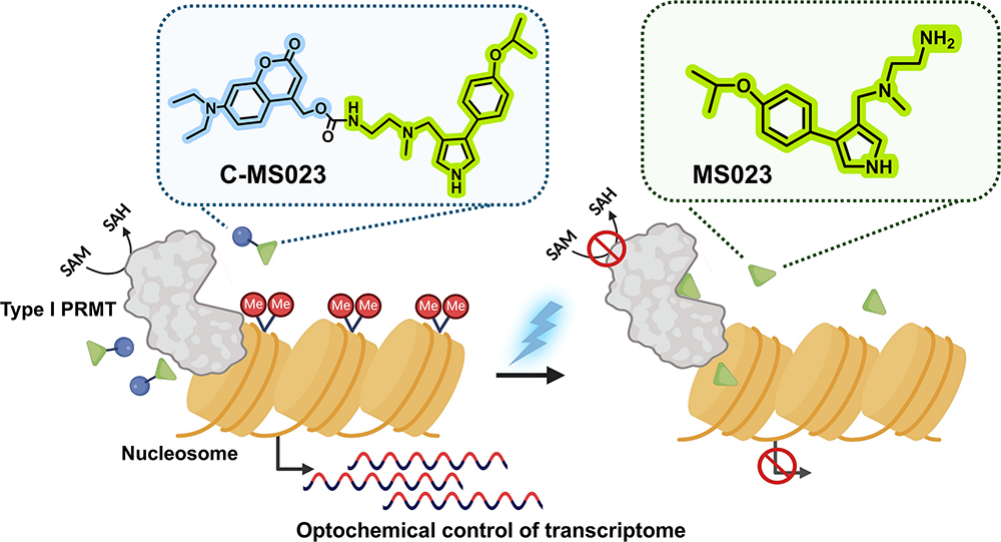
Schematic illustration of opto-epigenetic regulation of histone arginine asymmetric dimethylation via a photoactivatable prodrug (C-MS023) of a type I protein arginine methyltransferase (PRMT) inhibitor. Visible light irradiation at 420 nm triggers the photolysis of the C-MS023 prodrug to release the bioactive type I PRMT inhibitor MS023, which reverses the histone arginine asymmetric dimethylation status and represses DNA replication transcriptomes. SAM, S-adenosylmethionine; SAH, S-adenosylhomocysteine; Me, methyl group. The image was created by BioRender.com.

Inspired by the noninvasive properties and unsurpassed spatiotemporal controllability of light irradiation, there have been increasing efforts to explore light-controlled epigenetic phenomena using photoresponsive small-molecule prodrugs.^13−17^ Given the promise of the opto-epigenetic prodrug strategy for site-specific regulation of transcriptional events,^15,18,19^ we developed a novel photoactivatable prodrug to achieve visible light-controlled inhibition of histone arginine asymmetric dimethylation. MS023, a selective and potent inhibitor of type I PRMTs, was chosen to design such an opto-epigenetic prodrug. As shown in Figure 1, the prodrug (caged MS023, termed C-MS023) was designed by attaching the photocleavable moiety 7-(diethylamino)-4-(hydroxymethyl)-coumarin (DEACM) to the terminal amino group of MS023. The prodrug C-MS023 displayed significantly attenuated inhibitory effects on type I PRMTs, which are the predominant enzymes responsible for mammalian histone arginine asymmetric dimethylation. Upon light irradiation at 420 nm, the potency of C-MS023 was recovered due to the photocleavage of the protecting moiety and the release of bioactive MS023. This led to the downregulation of histone arginine asymmetric dimethylation and transcriptional silencing of cell cycle and DNA replication pathways in human lung adenocarcinoma A549 cells. This study demonstrated the potential of visible light-controlled inhibition of type I PRMTs as a targeted epigenetic therapeutic approach.

## 2 Results and Discussion

**Figure 2:**
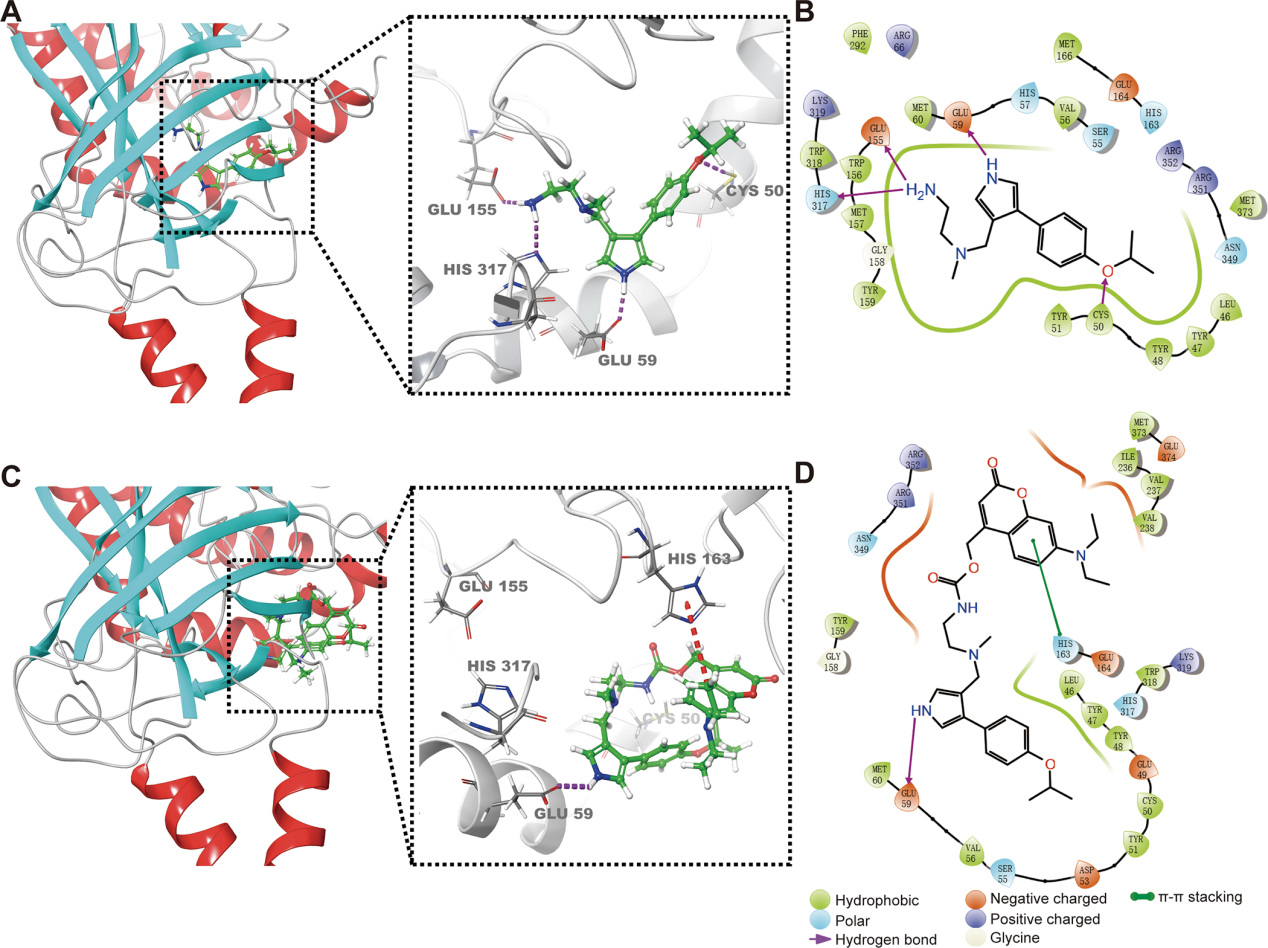
Molecular docking analysis of the interactions between PRMT6 (PDB code: 5E8R) and MS023 or C-MS023. (A) 3 D and (B) 2 D representation of docking results of MS023 with PRMT6. (C) 3 and (D) 2 D representation of docking results of C-MS023 with PRMT6. The hydrogen bonding and π–π stacking interactions are indicated by purple and red dashed lines for (A,C), respectively.

According to a previous report,^4^ ethylenediamine is a critical pharmacophore for the bioactivity of type I PRMT inhibitors. When the ethylenediamine moiety of MS023 was replaced with an aminoamide or hydroxyethylamino moiety, it exhibited no inhibition for type I PRMTs in vitro.^4^ Therefore, we hypothesized that masking the terminal primary amine group of MS023 with a photocaging moiety could prevent its binding to type I PRMTs. PRMT6 is an abundant type I PRMT enzyme in mammals, and it has been widely used for screening type I PRMT inhibitors.^4,9^ As a proof of concept, we first conducted molecular docking to investigate whether modifying the terminal amine group of MS023 with the photocleavable moiety DEACM would disturb the interactions between MS023 and PRMT6. As revealed in Figure 2A,B, the terminal amine group of MS023 formed hydrogen bonds with nearby amino acid residues of PRMT6, namely, Glu155 and His317 (purple dashed lines in Figure 2A). These hydrogen bonds have been recognized as the main contributors to the substrate arginine-binding affinity of MS023.^4,20^ In comparison, attaching the DEACM group to MS023 impaired the formation of these critical hydrogen bonds with the targeted residues of PRMT6 (Figure 2C,D). This may impede the occupation of C-MS023 in the substrate-binding pocket of PRMT6. These data are highly consistent with the published findings that replacing the primary terminal amine moiety with an amide substituent led to a significant reduction in the PRMT6 inhibitory efficacy of MS023.^4^

**Scheme 1:**
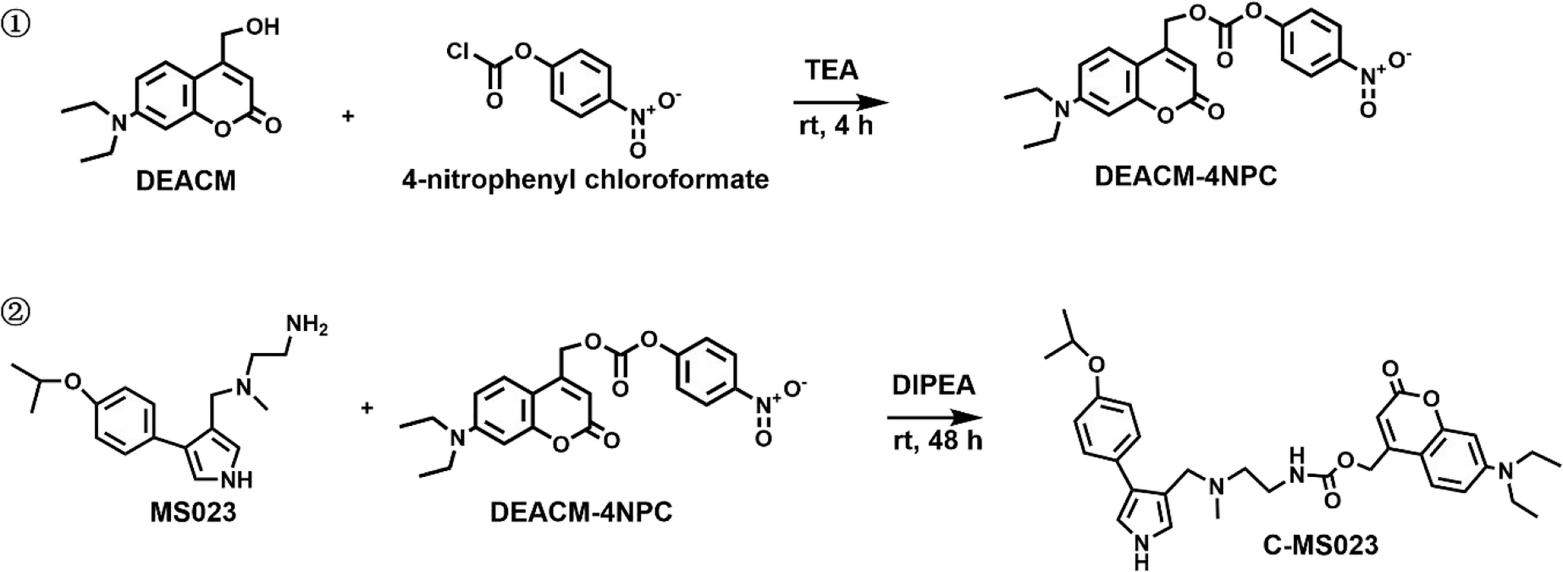
Synthesis route of C-MS023.

**Figure 3:**
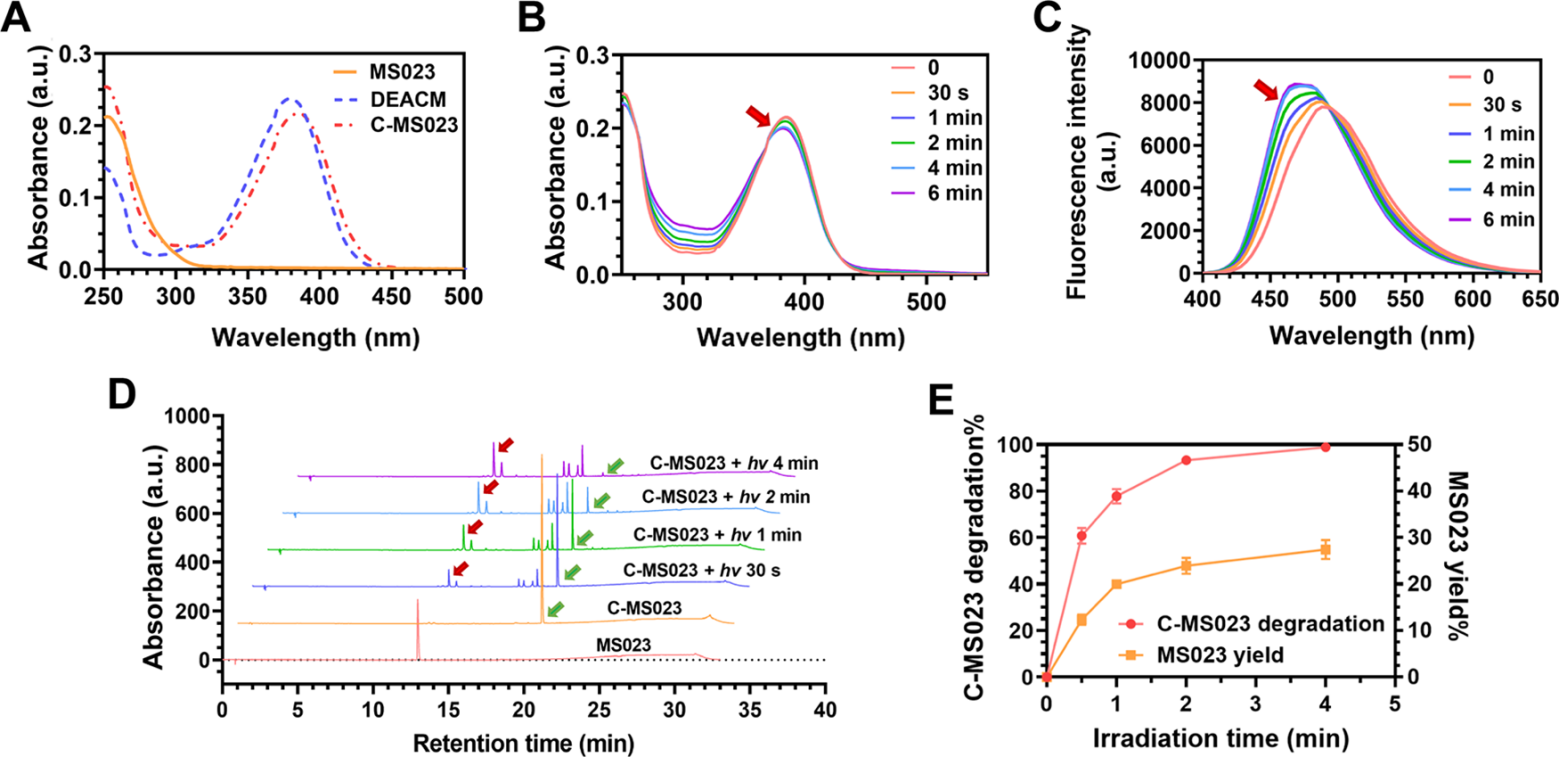
Photochemical characterization of C-MS023. (A) UV–vis absorption spectra of MS023, DEACM, and C-MS023. (B) UV–vis absorption spectra of C-MS023 upon visible light irradiation for varying periods (420 nm, 10 mW/cm^2^). (C) Fluorescence spectra of C-MS023 upon light irradiation for varying periods (420 nm, 10 mW/cm^2^). Ex = 385 nm. (D) Representative HPLC spectra and (E) quantitative results of C-MS023 after visible light irradiation (420 nm, 10 mW/cm^2^), showing the photolysis of the prodrug (denoted by green arrows) and the generation of MS023 (denoted by red arrows). The concentration of MS023, DEACM, and C-MS023 was set at 10 μM, with acetonitrile/water = 1:1 as the solvent. Data were presented as means ± SD (*n* = 3).

Subsequently, we synthesized the designed photoactivatable prodrug C-MS023 via a simple two-step reaction (Scheme 1). Its purity was tested by high-performance liquid chromatography (HPLC) analysis (Figure S1) with the mobile phase timetable detailed in Table S1. The chemical structure and molecular weight of C-MS023 were further validated by proton nuclear magnetic resonance (^1^H NMR) and mass spectroscopy (Figures S2 and S3). The obtained prodrug displayed similar absorption peaks to those of MS023 and DEACM (Figure 3A). We next investigated the photochemical performance of C-MS023 upon visible light irradiation at 420 nm. As shown in Figure 3B,C, after light irradiation, a slight hypsochromic shift was observed at the maximum absorption peak of C-MS023, from 385 to 380 nm. Additionally, the maximum fluorescence emission wavelength of C-MS023 shifted from 490 to 470 nm after light irradiation. These phenomena are comparable to the photophysical properties of the photolysis product DEACM (Figures 3A and S4), confirming that photolysis of C-MS023 occurred. Moreover, HPLC data revealed that 420 nm light irradiation led to a gradual rise in the peak of MS023, coincident with a significant decrease in the intensity of the C-MS023 peak (Figure 3D,E). Visible light irradiation (420 nm, 10 mW/cm^2^) for 4 min resulted in the almost disappearance of C-MS023, enabling efficient light-controlled inhibition of type I PRMTs at a relatively low irradiation dose. The maximum phototriggered release yield of MS023 from the prodrug was about 27.4% (420 nm, 10 mW/cm^2^, 4 min). It is widely recognized that heterolytic bond cleavage is the primary photolysis mechanism of (coumarin-4-yl)methyl carbamates, followed by the hydrolysis and decarboxylation process to release amine-terminated compounds.^21−23^ The proposed photolysis mechanism of C-MS023 is shown in Figure S5. During this process, undesirable recombination byproducts could be generated.^24,25^ The incomplete release of coumarin-caged molecules was also demonstrated by the previous reports.^22,26,27^ Meanwhile, more than 75% of C-MS023 remained in pH 7.4 PBS buffer at 37 °C for 72 h without light exposure, suggesting its excellent dark stability in vitro (Figure S6).

**Figure 4:**
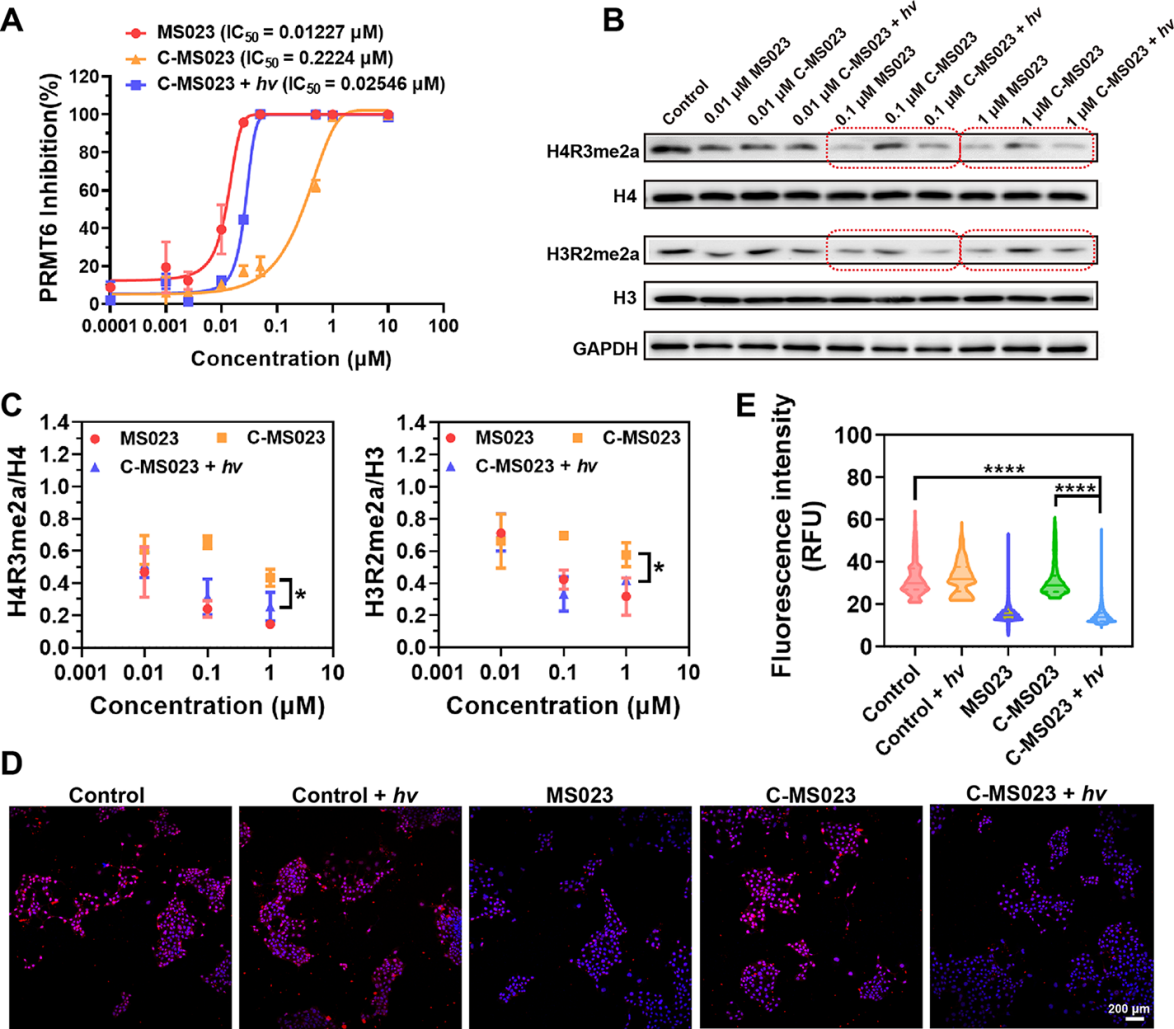
In vitro visible light-triggered inhibition of histone arginine asymmetric dimethylation with C-MS023. (A) Concentration-PRMT6 inhibition curves of MS023 and C-MS023 with light irradiation (420 nm, 10 mW/cm^2^, 4 min) or not. The methyltransferase activity kit was used to assess the effect of the indicated drugs on PRMT6 activity (*n* = 3). (B) Representative Western blot images and (C) quantitative results of histone arginine asymmetric dimethylation levels in A549 cells after different treatments. A549 cells were incubated with varying concentrations of MS023 or C-MS023 for 24 h, followed by light irradiation (420 nm, 10 mW/cm^2^, 4 min) or not. The cells were cultured for another 24 h before detection of H4R3me2a and H3R2me2a levels by Western blot (*n* = 3). (D) Representative confocal fluorescence images and (E) quantitative results of H4R3me2a immunofluorescence staining of A549 cells after different treatments. A549 cells were treated with MS023 or C-MS023 (0.1 μM) for 24 h with or without light irradiation (420 nm, 10 mW/cm^2^, 4 min) (*n* = 6). Red, H4R3me2a-positive nuclei; Blue, DAPI-stained nuclei. *p < 0.05; ****p < 0.0001.

To investigate the impact of visible light irradiation on the type I PRMT inhibitory efficacy of C-MS023, we performed the methyltransferase activity assay with PRMT6 and histone 3 (H3) as the substrate, as described in the previous literature.^28^ As shown in Figure 4A, MS023 exhibited robust inhibition of PRMT6 activity. Its estimated 50% inhibitory concentration (IC_50_) was 0.01227 μM, similar to the previous report.^4^ In contrast, we found that C-MS023 was less effective in inhibiting PRMT6-mediated asymmetric dimethylation of H3 arginine 2 (H3R2me2a), with an estimate IC_50_ of 0.2224 μM, 18 times higher than that of MS023. Notably, the C-MS023 plus light irradiation group displayed a comparable potency for PRMT6 inhibition as MS023 treatment, with an estimate IC_50_ of 0.02546 μM. These results indicate that 420 nm light irradiation could cause the photouncaging of C-MS023 and the release of the desired product to inhibit histone arginine asymmetric dimethylation.

Moreover, we explored the possibility of photocontrolled downregulation of histone arginine asymmetric dimethylation in live cells with this opto-epigenetic prodrug. The expression of type I PRMTs is reported to be preferentially upregulated in several human cancers, such as non-small-cell lung cancer.^29,30^ With these considerations, human lung adenocarcinoma cell line A549 was chosen for in vitro studies. Additionally, A549 cells were also most susceptible to MS023-mediated type I PRMT inhibition among the tested tumor cell lines (Figure S7). As histone methylation modifications predominantly occur at histone 3 (H3) and histone 4 (H4),^31^ we examined the levels of asymmetric dimethylation at histone 3 arginine 2 (H3R2me2a) and histone 4 arginine 3 (H4R3me2a) by Western blot analysis. As shown in Figure 4B,C, MS023 treatment resulted in a remarkable reduction of H3R2me2a and H4R3me2a marks in A549 cells at concentrations as low as 0.1 μM. In comparison, the designed prodrug showed weak influence on cellular levels of H3R2me2a and H4R3me2a at concentrations up to 1 μM, whereas those protein levels could be significantly downregulated after 420 nm light irradiation. Overall, these results validate the photoactivated type I PRMT inhibitory effect of C-MS023.

To visualize the changes in histone arginine asymmetric dimethylation levels in vitro, immunofluorescence staining of H4R3me2a was performed after A549 cells were treated with MS023 and C-MS023 with or without light exposure. As shown in Figure 4D,E, the red fluorescence intensity indicating H4R3me2a levels in C-MS023-treated cells was comparable to that of the control. However, red fluorescence intensity dramatically dropped in the C-MS023 plus light irradiation group, similar to that of the MS023 group. These findings are consistent with the Western blot results, indicating that C-MS023 exhibited limited PRMT inhibitory capacity and light-induced recovery of its potency.

**Figure 5:**
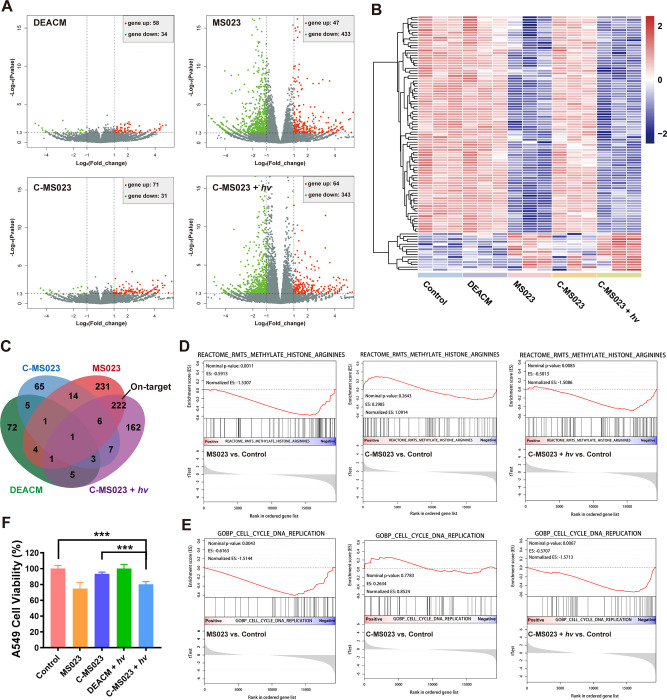
Characterization of the photocontrolled alterations of transcriptomic profiles with C-MS023 in vitro. (A) Volcano plots of log2 fold change of genes significantly upregulated (red, right) or downregulated (green, left) after treatments with the indicated drugs. A549 cells were incubated with 0.1 μM of DEACM, MS023, or C-MS023 for 24 h, followed by light irradiation (420 nm, 10 mW/cm^2^, 4 min) or not. The cells were cultured for another 24 h before the transcriptomic analysis. (B) Heatmap showing the top 50 differentially expressed genes after A549 cells were treated with the indicated drugs. (C) Venn diagram showing the overlap of differentially expressed genes among various groups. (D) Gene set enrichment analysis (GSEA) of the gene sets associated with PRMT-mediated histone arginine methylation. tTest parameter was used as the metric for ranking genes. (E) GSEA of the gene sets associated with the cell cycle and DNA replication. (F) A549 cell viability after treatments with the indicated drugs. A549 cells were incubated with 10 μM of DEACM, MS023, or C-MS023 for 24 h, followed by light irradiation (420 nm, 10 mW/cm^2^, 4 min) or not. The cells were cultured for another 48 h before MTT assay (*n* = 6). ***p < 0.001.

Furthermore, we investigated the changes in transcription signatures of C-MS023-treated A549 cells by RNA-sequencing analysis. We found that neither C-MS023 treatment alone nor DEACM treatment induced significant alterations in the transcriptomic profiles of A549 cells (Figure 5A). Interestingly, C-MS023 plus light irradiation led to remarkable transcriptomic changes, with an ∼5.4:1 ratio between the downregulated and upregulated genes observed. These phenomena are in line with the previous reports that type I PRMT ablation is mainly linked to transcriptional repression, despite some exceptions.^3,32^ Heatmap visualization of rank-ordered genes revealed similar differential expression patterns between C-MS023 plus light irradiation and MS023 (Figure 5B). Approximately 56% of the differentially expressed genes in the C-MS023 plus light irradiation group overlapped with those in the MS023 group (Figure 5C). Gene set enrichment analysis (GSEA) also demonstrated a prominent reduction of PRMT-induced histone arginine methylation activity for C-MS023-treated cells with light irradiation (Figure 5D). In contrast, we did not observe such obvious downregulation of gene sets related to histone arginine methylation in C-MS023 treatment alone. Taken together, these results suggest that on-target inhibition of type I PRMT activity with C-MS023 could be achieved by modulation of light irradiation.

The tumor growth-inhibitory properties of type I PRMT inhibitor MS023 have been demonstrated in multiple cancer cell lines, with a concomitant decline in asymmetric arginine dimethylation.^6,10,33^ Inspired by previous research, we further investigated the light-dependent antiproliferative activity of C-MS023 in vitro. As shown in Figure 5E, pathways related to the cell cycle and DNA replication were not significantly downregulated in the C-MS023 group, but a strong downregulation of these pathways was observed in the MS023 and C-MS023 plus light irradiation groups. Additionally, A549 cell proliferation was slowed down after 48 h of treatment with C-MS023 plus light irradiation (79.4% of cell viability), presumably owing to the phototriggered release of MS023 (Figure 5F). In comparison, C-MS023 or DEACM plus light irradiation showed negligible impacts on A549 cell viability (94.6–101.0% of cell viability). Meanwhile, C-MS023 did not show apparent cytotoxicity to human umbilical vein endothelial cells at a relatively high dose (50 μM) (Figure S8). These findings indicate the effectiveness and safety of C-MS023 as a photocontrolled epigenetic drug for antitumor research.

## 3 Conclusions

In this study, we developed a novel type I PRMT inhibitor prodrug, C-MS023, which enables controllable inhibition of histone arginine asymmetric dimethylation via visible light irradiation. The prodrug was designed by employing the photocleavable group DEACM to mask the key pharmacophore of MS023. We validated that the C-MS023 prodrug possessed little inhibitory effect on type I PRMT activity in the dark. However, upon exposure to a relatively low irradiation dose (420 nm, 10 mW/cm^2^, 4 min), the prodrug showed potent activity in inhibiting histone arginine asymmetric dimethylation in vitro. This method may allow for the localized release of MS023 upon light irradiation at the tumor site, without affecting the broad physiological activities of type I PRMTs elsewhere. Moreover, transcriptomic assay results demonstrate the feasibility of light-induced type I PRMT inhibition for specifically downregulating DNA replication pathways in tumor cells. The success of this study provides proof of concept for the opto-epigenetic prodrug strategy to achieve spatiotemporal regulation of type I PRMT activities, potentially assisting in the future development of epigenetic therapeutics for precise antitumor treatment. To overcome the common limitations of superficial tissue penetration of visible light, the use of upconverting nanoparticles to convert near-infrared light to short-wavelength light or the utilization of optical fibers or implantable light-emitting diodes (LEDs) may be a potential direction to facilitate the in vivo applications of this prodrug.

## 4 Experimental Section

### 4.1 Materials and Instruments

MS023 was purchased from MedChemExpress (New York, USA). 4-Nitrophenyl chloroformate, 7-(diethylamino)-4-(hydroxymethyl)-coumarin (DEACM), 3-(4,5-dimethylthiazol-2-yl) 2,5-diphenyl tetrazolium bromide (MTT), *N,N*-diisopropylethylamine (DIPEA), and triethylamine (TEA) were obtained from Macklin Biochemical Co., Ltd. (Shanghai, China). The methyltransferase activity assay kit (colorimetric) (ab273307), recombinant human Histone H3 protein (ab198757), and recombinant human PRMT6 protein (ab268890) were obtained from Abcam Co., Ltd. (Cambridge, U.K.). All chemicals used were of analytical or ACS reagent grade. Chemical purity was >95%, as confirmed by HPLC analysis (Figure S1).

Product purification and detection were conducted with an Agilent 1260 Infinity II HPLC system. The proton nuclear magnetic resonance (^1^H NMR) spectrum was obtained by a Bruker AVANCE III 600 MHz NMR spectrometer, with CDCl_3_ as the deuterated solvent. The mass spectrum was obtained by a Waters AutoPurification LC/MS system. UV–vis absorption and fluorescence spectra were acquired by a SpectraMax M4 multimode microplate reader. Mightex collimated LED (420 nm) was used as the light source for irradiation procedures. Light irradiance was detected by a THORLABS PM100USB optical power meter with an S142C sphere photodiode power sensor before use.

### 4.2 Molecular Docking

The cocrystal structures of PRMT6 in complex with MS023 (PDB code: 5E8R) were retrieved from the Protein Data Bank and imported into Maestro 11.5 software (Schrödinger, Inc., USA). After removal of water and ligands, receptor preparation of PRMT6 was processed by the Protein Preparation Wizard with standard settings, including adding hydrogen, fixing chain bonds, optimizing protonation states, and energy minimization by the OPLS3 force-field. The protein grid was generated by picking the surrounding residues of cocrystallized MS023 with the Receptor Grid Preparation tool. The ligand structures of MS023 and C-MS023 were prepared by the LigPrep function, wherein diverse conformations of the ligands were generated including the tautomeric states. Then, ligand docking was performed using default parameters with the XP (precise precision) mode. The top-scored docking poses of PRMT6 in complex with the ligands were visualized by the software workspace and Ligand Interaction Diagram function. The PDB files and the atomic coordinate information are provided in the Supporting Information.

### 4.3 Synthesis of C-MS023

The synthetic routes of C-MS023 are shown in Scheme 1 according to the previous studies.^34,35^ First, the photocaging group 7-(diethylamino)-4-(hydroxymethyl)-coumarin (DEACM) (0.8 mmol, 1 equiv) and 4-nitrophenyl chloroformate (3.2 mmol, 4 equiv) were mixed in a dry and nitrogen-backfilled flask. Anhydrous DCM (1 mL) was added to dissolve the reactants. Then, triethylamine (TEA) (3.2 mmol, 4 equiv) was added and stirred for 4 h at room temperature to activate the DEACM to DEACM-carbonate (DEACM-4NPC). The reactions were monitored by thin-layer chromatography, and the mixtures were purified by column chromatography. The yield of DEACM-4NPC was ∼72%.

The resulting DEACM-4NPC (0.5 mmol, 1 equiv) was next reacted with MS023 (0.5 mmol, 1 equiv) in the presence of *N,N*-diisopropylethylamine (DIPEA) (1 mmol, 2 equiv) in anhydrous DCM (1 mL) for 48 h. The final product was purified by the HPLC system equipped with a C18 column (Agilent ZORBA SB-C18, 9.4 × 250 mm), with a yield rate of ∼56%. The chemical structure of C-MS023 was confirmed by ^1^H NMR spectroscopy.

### 4.4 Photochemical Characterization and Photolysis Assessment

The UV–vis absorption spectra of MS023, C-MS023, and DEACM (10 μM) were detected by a multimode microplate reader. The changes in the UV–vis absorption and fluorescence spectra of C-MS023 were also characterized upon 420 nm light irradiation for varying periods. Additionally, 10 μM of C-MS023 solutions in a mixed solvent of water and acetonitrile (1:1) was exposed to 420 nm light irradiation (10 mW/cm^2^) for 0, 0.5, 1, 2, and 4 min, followed by HPLC analysis of the remaining C-MS023 and the released MS023. The HPLC conditions were listed as follows: an Agilent InfinityLab Poroshell 120 EC-C18 column (2.7 μm, 100 mm × 4.6 mm) with a Poroshell 120 UHPLC Guard column; using a gradient dilution method with acetonitrile with 0.1% trifluoroacetate (TFA) and water with 0.1% TFA; a column temperature of 25 ± 2 °C; and a detection wavelength of 254 or 420 nm. The mobile phase timetable is listed in Table S1.

### 4.5 Methyltransferase Activity Assay

The methyltransferase inhibition experiment was performed following the manufacturer’s protocol (ab273307, Abcam). Briefly, 50 nM PRMT6 buffer was preincubated with a series concentrations of MS023 or C-MS023 for 10 min. Then, the methyltransferase reaction buffer containing 0.6 μM human histone H3 protein was added to the mixtures, followed by the addition of a 1× detection solution. After incubation at room temperature for 30 min, precooled isopropanol was added to the reactants. The fluorescence intensity was detected at an excitation/emission wavelength of 380 nm/520 nm. 2.3 μM of S-(5′-adenosyl)-l-homocysteine solutions was used as the positive control.

### 4.6 Cell Culture and Cell Viability Detections

Cells were grown in Dulbecco’s modified Eagle’s medium (DMEM) supplemented with 10% (w/v) FBS and 1% (w/v) penicillin–streptomycin. The parameters of the cell incubator were set at 37 °C and 5% CO_2_.

To evaluate the cytotoxicity effects of MS023 or C-MS023, the indicated cells were cultured on 96-well plates at a seeding density of 4 × 10^3^ cells/well overnight. Then, the cells were treated with the culture medium containing varying concentrations of MS023 or C-MS023 for 24 h. Subsequently, the cells were exposed to light irradiation (420 nm, 10 mW/cm^2^, 4 min) or not and cultured for 48 h. Then, the cell culture medium was replaced with fresh medium containing 0.5 mg/mL of (3-(4,5-dimethylthiazol-2-yl)-2,5-diphenyltetrazolium bromide) (MTT). After 4 h of incubation, the culture medium was removed, and 100 μL of DMSO was added into each well to fully dissolve the formazan crystals. The absorbance of the 96-well-plate was detected by a microplate reader at 570 nm. Cell viability was calculated as a percentage of the control.

### 4.7 Western Blot Assay and Transcriptomic Analysis

To detect the alterations in the histone arginine asymmetric dimethylation levels, A549 cells were grown on 6-well plates at a seeding density of 5 × 10^4^ cells/well and cultured overnight. The cells were incubated with varying concentrations of MS023 or C-MS023 (0∼1 μM) for 24 h, then exposed to light irradiation (420 nm, 10 mW/cm^2^, 4 min), or not. The cells were cultured for another 24 h. After different treatments, cells were lysed with RIPA buffer containing 1% protease inhibitor cocktail (P8340, Merck) and incubated on ice for 30 min. After centrifugation at 12,000 g, 4 °C for 15 min, total protein samples were attained by collecting the supernatants, and the protein concentrations were detected by the Pierce BCA Protein Assay Kit (23225, ThermoFisher). Western blot analysis of the normalized protein samples was conducted following the generally established protocols. The antibodies used in this study were as follows: asymmetric dimethyl-histone H3-R2 antibody (1:1000 dilution, A3155, ABclonal), asymmetric dimethyl-histone H4-R3 antibody (1:1000 dilution, A2376, ABclonal), histone H3 antibody (1:1000 dilution, A2348, ABclonal), histone H4 antibody (1:1000 dilution, A8466, ABclonal), and goat anti-rabbit IgG H&L (HRP) (1:2000 dilution, ab205718, Abcam).

For sample preparation of high-throughput RNA-sequencing (RNA-seq), A549 cells (5 × 10^6^ cells/dish) were treated with 0.1 μM of MS023 or C-MS023 for 24 h and received light irradiation (420 nm, 10 mW/cm^2^, 4 min) or not. After incubation for 24 h, the cells were gently washed with PBS buffer three times and subjected to RNA isolation by TRIzol reagent. RNA-seq sample processing and data analysis were carried out by Ribobio Co. Ltd. (Guangzhou, China). Quality control analysis of RNA-seq samples was performed with an Agilent 2200 TapeStation system, and library sequencing was conducted by paired-end 150 bp Illumina sequencing. Log fold change greater than 1 (|log_2_FC| > 1) and adjusted p-values smaller than 0.05 (adj. *P* < 0.05) based on the Benjamini and Hochberg test correction method were screened of raw count data as differential expression genes. Gene set enrichment analysis (GSEA) was conducted by GSEA 4.3.2 software (UC San Diego and Broad Institute, USA) with the Human MSigDB v2023.1 Database. Signature gene sets (REACTOME_RMTS_METHYLATE_HISTONE_ARGININES, GOBP_CELL_CYCLE_DNA_REPLICATION) were used for the analysis of histone arginine methylation and DNA replication transcriptomes.

### 4.8 Immunofluorescent Staining

To visualize the H4R3me2a levels after different treatments, A549 cells were plated on sterile confocal dishes at a seeding density of 2 × 10^4^ cells/dish and cultured overnight. Then, the cells were treated with 0.1 μM MS023 or C-MS023 for 24 h, followed by light irradiation (420 nm, 10 mW/cm^2^, 4 min) or not. The cells were cultured for another 24 h and washed with PBS buffer for three times. After fixation and permeabilization, the cells were blocked with 5% bovine serum albumin (BSA) for 1 h and incubated with asymmetric dimethyl-histone H4-R3 antibody (1:200 dilution, A2376, ABclonal) overnight at 4 °C. After several washes with PBS buffer, the cells were incubated with goat anti-rabbit IgG H&L Alexa Fluor-594 (1:500 dilution, ab150080, Abcam) for 1 h and stained by 10 μg/mL DAPI for 15 min. The cells were viewed under a ZEISS LSM 900 confocal microscope (Leica, Germany). The red fluorescence intensity of H4R3me2a-positive nuclei was further quantified by ImageJ software.

### 4.9 Statistical Analysis

Statistical analysis was completed with GraphPad Prism version 9.4.0 software. Data were presented as mean ± SD (standard deviation) (*n* = 3), except for specific notifications. Two-tailed unpaired *t* test or two-way ANOVA with Bonferroni’s post hoc test was conducted for between-group analysis or comparison of more than two groups. *p* values <0.05 were recognized statistically significant.

## Data Availability

The data supporting the findings of this study are available on request from the corresponding author.

## Supporting Information Available

High-performance liquid chromatography (HPLC) analysis of chemical purity and prodrug stability; mobile phase timetable of HPLC analysis; proton nuclear magnetic resonance (^1^H NMR) spectra of synthesized compounds; mass spectrum of C-MS023; fluorescence spectrum of DEACM; proposed photolysis mechanism of C-MS023; and cell viability assay with MS023 (PDF)
Table of molecular formula strings (CSV)
Docking of MS023 with PRMT6 (PDB ID:5E8R) (PDB)
Docking of C-MS023 with PRMT6 (PDB ID:5E8R) (PDB)

## Supplementary Material

jm4c02199_si_002.csv

jm4c02199_si_003.pdb

jm4c02199_si_004.pdb
