# Supplementary material for: Opto-Epigenetic Regulation of Histone Arginine Asymmetric Dimethylation via Type I Protein Arginine Methyltransferase Inhibition
Source: J Med Chem. 2025 Feb 17;68(4):4373–81. doi: 10.1021/acs.jmedchem.4c02199 (PMC11873949; doi:10.1021/acs.jmedchem.4c02199)
Supplement: Supplementary file 1 — jm4c02199_si_001.pdf [file jm4c02199_si_001.pdf]

Supporting information

Opto-epigenetic regulation of histone arginine asymmetric dimethylation via type I protein arginine methyltransferase inhibition

Shuting Xu[1, 2, 3], *Kaiqi Long*[1, 2, 3], *Tianyi Wang*[1, 2, 3], *Yangyang Zhu*[4, 5], *Yunjiao Zhang*[4, 5], *Weiping Wang*[*1, 2, 3]

1 State Key Laboratory of Pharmaceutical Biotechnology, The University of Hong Kong, Hong Kong, China.

2 Department of Pharmacology and Pharmacy, Li Ka Shing Faculty of Medicine, The University of Hong Kong, Hong Kong, China.

3 Laboratory of Molecular Engineering and Nanomedicine, Dr. Li Dak-Sum Research Centre, The University of Hong Kong, Hong Kong, China.

4 The Second Affiliated Hospital, School of Medicine, South China University of Technology, Guangzhou, 510006 P. R. China.

5 School of Biomedical Sciences and Engineering, National Engineering Research Center for Tissue Restoration and Reconstruction and Key Laboratory of Biomedical Engineering of Guangdong Province, South China University of Technology, Guangzhou, 510006 P. R. China.

*Corresponding author, Prof. W. Wang, E-mail address: wangwp@hku.hk

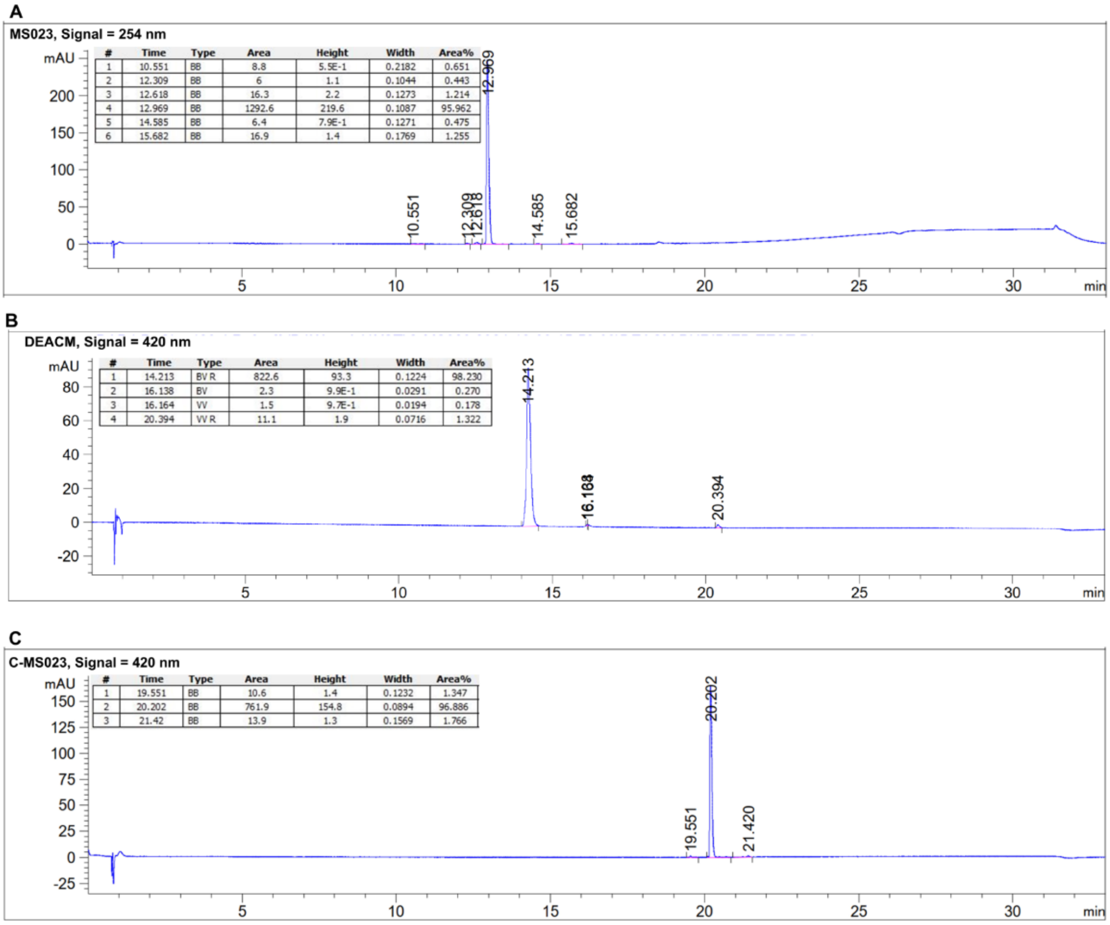

#	Time	Type	Area	Height	Width	Area%
1	10.551	BB	8.8	5.5E-1	0.2182	0.651
2	12.309	BB	6	1.1	0.1044	0.443
3	12.618	BB	16.3	2.2	0.1273	1.214
4	12.969	BB	1292.6	219.6	0.1087	95.962
5	14.585	BB	6.4	7.9E-1	0.1271	0.475
6	15.682	BB	16.9	1.4	0.1769	1.255

#	Time	Type	Area	Height	Width	Area%
1	14.213	BV R	822.6	93.3	0.1224	98.230
2	16.138	BV	2.3	9.9E-1	0.0291	0.270
3	16.164	VV	1.5	9.7E-1	0.0194	0.178
4	20.394	VV R	11.1	1.9	0.0716	1.322

#	Time	Type	Area	Height	Width	Area%
1	19.551	BB	10.6	1.4	0.1232	1.347
2	20.202	BB	761.9	154.8	0.0894	96.886
3	21.42	BB	13.9	1.3	0.1569	1.766

Figure S1. Representative high-performance liquid chromatography (HPLC) graphs for purity analysis of (A) MS023, (B) DEACM, (C) C-MS023 for cell experiments.

Table S1. Mobile phase timetable of high-performance liquid chromatography (HPLC) analysis. Mobile phase A: acetonitrile with 0.1% trifluoroacetate (TFA); Mobile phase B: water with 0.1% TFA.

Time (min)	A (%)	B (%)	Flow (mL/min)
0.0	5.0	95.0	1.5
3.0	5.0	95.0	1.5
10.0	20.0	80.0	1.5
17.0	40.0	60.0	1.5
22.0	80.0	20.0	1.5
25.0	100.0	0.0	1.5
30.0	100.0	0.0	1.5
33.0	5.0	95.0	1.5

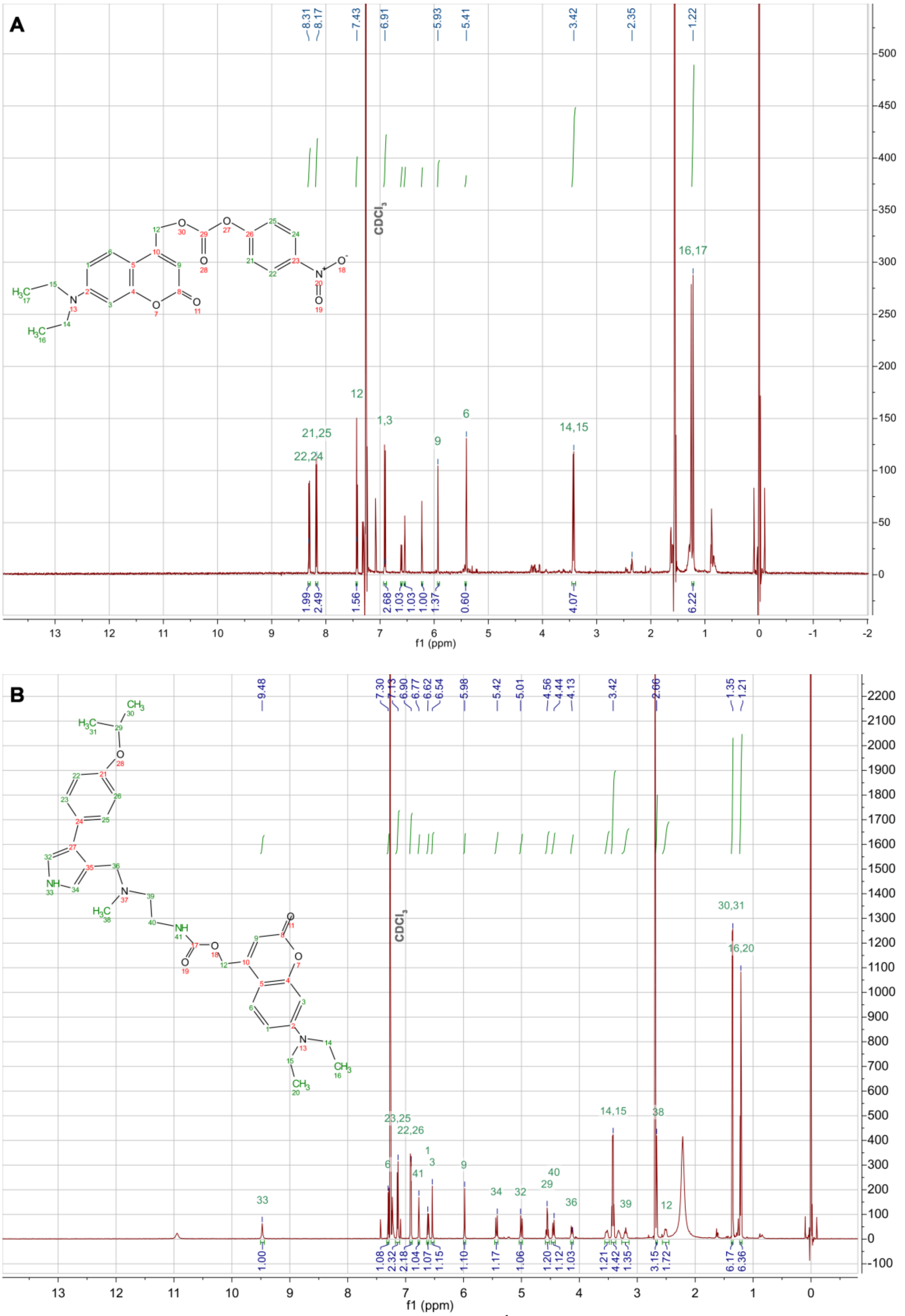

Figure S2. Nuclear magnetic resonance hydrogen (^{1}H NMR) spectra of (A) DEACM-4NPC and (B) C-MS023.

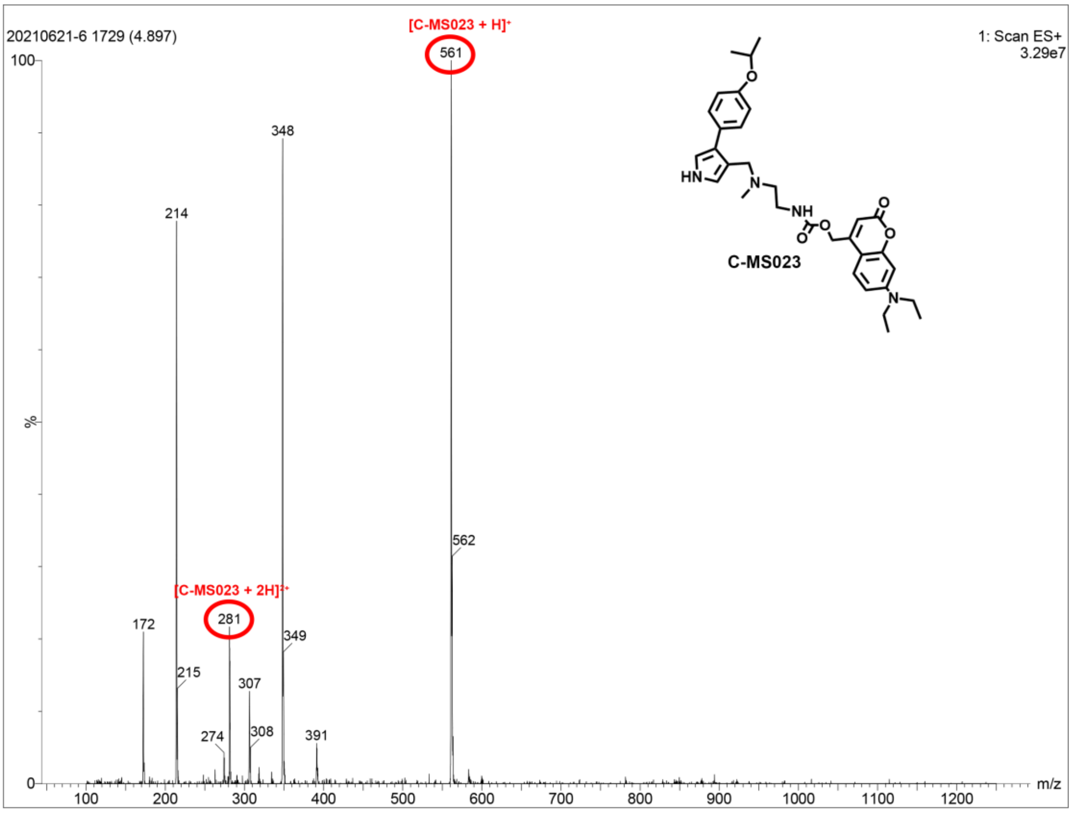

Figure S3. Mass spectrum of C-MS023.

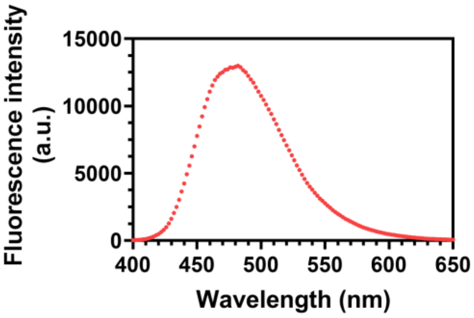

Figure S4. Fluorescence spectrum of DEACM. Ex = 385 nm. The concentration of DEACM was set at 10 µM, acetonitrile/water = 1:1 as the solvent.

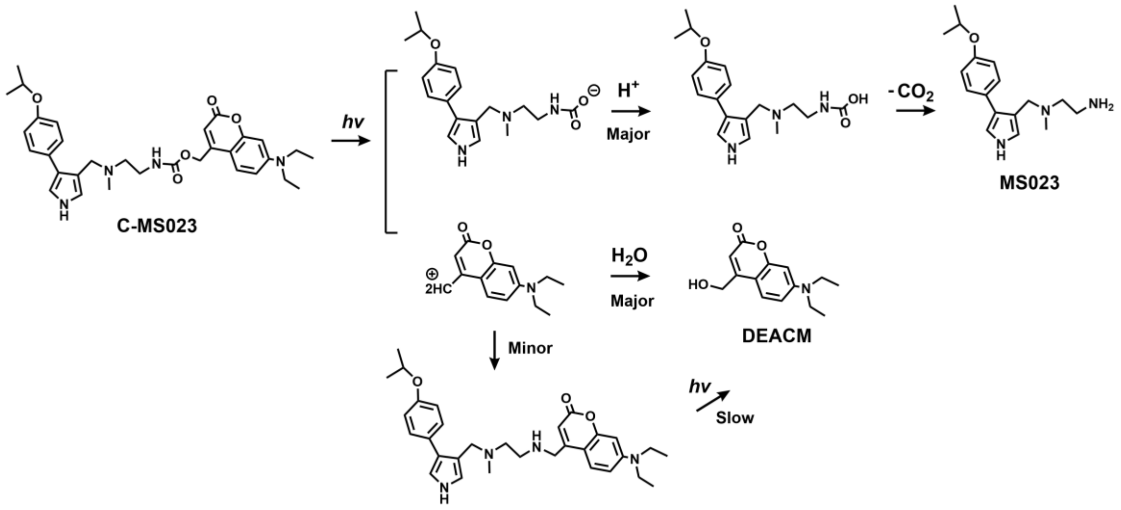

Figure S5. Schematic illustration of proposed photolysis mechanism of C-MS023.

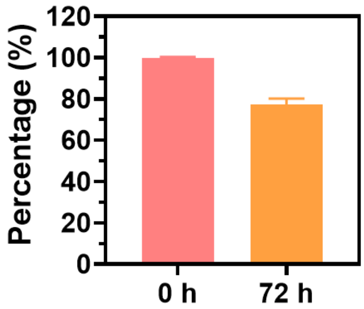

Figure S6. HPLC quantitative analysis of C-MS023 before and after incubation in pH7.4 PBS buffer at 37 °C for 72 h. Data were presented as mean ± standard deviations (n = 3).

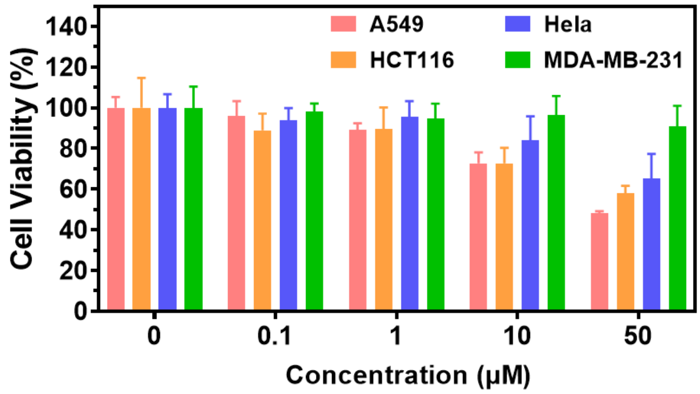

Figure S7. Cell viability of different cell lines treated with varying concentrations of MS023 for 72 h. A549, human lung adenocarcinoma cell line; HCT116, human colorectal carcinoma cell line; Hela, human cervical adenocarcinoma cell line; MDA-MB-231, human breast cancer cell line. Data were presented as mean ± standard deviations (n = 6).

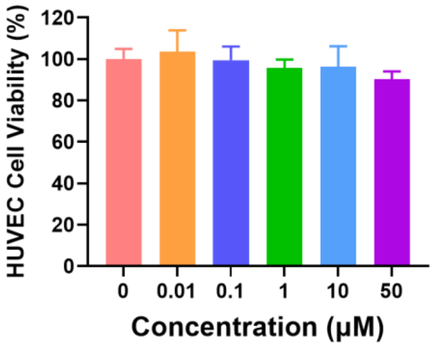

Figure S8. Cell viability of human umbilical vein endothelial cells (HUVECs) treated with varying concentrations of C-MS023 for 72 h in the dark. Data were presented as mean ± standard deviations (n = 6).
